# Exploring the role and mechanisms of MAGEA4 in tumorigenesis, regulation, and immunotherapy

**DOI:** 10.1186/s10020-025-01079-8

**Published:** 2025-02-04

**Authors:** Weijian Zhu, Qiang Yi, Zheng Chen, Jiaqi Wang, Kui Zhong, Xinting Ouyang, Kuan Yang, Bowei Jiang, Jianing Zhong, Jinghua Zhong

**Affiliations:** 1 Gannan Medical University, Ganzhou, Jiangxi China; 2 Department of Oncology, First Affiliated Hospital of Gannan Medical University, Ganzhou, Jiangxi China

**Keywords:** MAGEA4, Tumorigenesis, Mechanisms, Immunotherapy

## Abstract

MAGEA4 is a member of the Melanoma-Associated Antigen (MAGE) family, characterized by high expression in various tumor tissues but low expression in normal tissues, with the exception of testis and placenta. Its expression is associated with poor prognosis in cancer. This review summarizes the mechanisms of action, regulatory functions, and immunotherapeutic applications of MAGEA4 in cancer.MAGEA4 promotes tumor initiation and progression through multiple pathways, including ubiquitination and degradation of the tumor suppressor P53, regulation of cell cycle and apoptosis, modulation of DNA damage repair, and enhancement of cancer cell survival. By forming a complex with TRIM28, MAGEA4 accelerates tumor development via P53 degradation. Factors such as TWIST1 and BORIS can upregulate MAGEA4 expression. MAGEA4 interacts with proteins including Miz-1, p53, and RAD18, participating in gene transcription regulation and DNA damage repair. By stabilizing RAD18, MAGEA4 facilitates the recruitment of Y-family DNA polymerases, enabling cells to continue replication under DNA damage conditions and thus supporting cancer cell survival. MAGEA4-based TCR-T cell therapy and cancer vaccines show clinical potential. This article comprehensively reviews the structure and function of MAGEA4, as well as recent research progress in solid tumors, providing a theoretical foundation for the clinical translation of MAGEA4 and its application in immunotherapy.

## Introduction

**Fig. 1:**
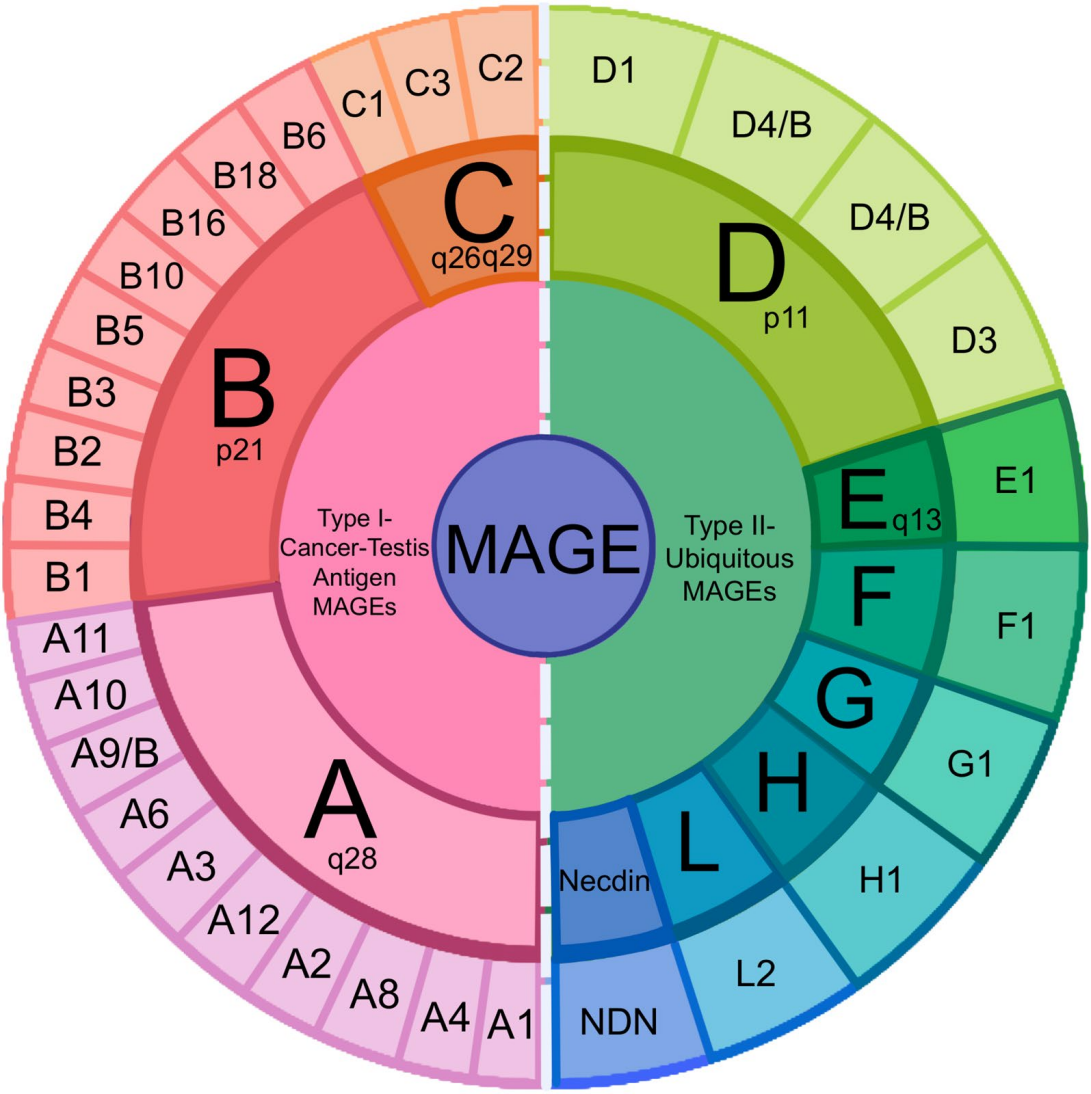
Distribution of Human MAGE Protein Family on the X Chromosome. (Lee and Potts 2017)

Recent data reveal that out of 19.37 million new cancer cases globally, 4.72 million (24.9%) occur in China. Similarly, of the 9.98 million cancer-related deaths worldwide, 2.57 million (25.8%) are reported in China, placing it at the forefront globally (Han et al. 2024). Cancer poses a significant burden on global public health, necessitating the urgent development of novel immunotherapeutic targets to improve treatment efficacy and patient prognosis. The Melanoma Antigen (MAGE) family, widely expressed in tumors and associated with poor prognosis (Weon and Potts 2015), represents a potential avenue for such development. The MAGE family comprises several subfamilies, including MAGE-A, MAGE-B, and MAGE-C (Fig. 1). The MAGE-A subfamily, the first tumor-associated antigen identified at the molecular level, exhibits consistent expression across most human cancers and germ cells. MAGE proteins (e.g., MAGE-A1, A3, A4, A6, A10, A12, C2) have been utilized as immunotherapeutic targets in numerous cancer clinical trials. MAGE-A proteins are associated with the activity of E3 RING ubiquitin ligases and can interact directly with the p53 tumor suppressor or indirectly regulate cancer cell survival processes by modulating E3 RING ubiquitin ligase activity. For instance, MAGE-A can bind to TRIM28, enhancing the activity of E3 ubiquitin ligase, which marks the tumor suppressor p53 for proteasomal degradation, thereby promoting tumorigenesis (Lee and Potts 2017). The aberrant expression of the MAGE-A family is linked to cancer cell proliferation, survival, and resistance to various therapies, making them ideal biomarkers and attractive therapeutic targets. Therefore, developing novel therapies targeting the MAGE-A family represents a promising research direction.

Melanoma-associated antigen 4 (MAGEA4, also known as MAGE-A4 or MAGE4) is a tumor/testis antigen belonging to the MAGE-A protein family subtype. The MAGE-A subfamily is primarily located in the q28 region of the X chromosome, spanning approximately 45 kb and consisting of 3 exons. The first two exons are relatively short, while the third is considerably longer. MAGE-A encodes a protein of 309–319 amino acids (Lee and Potts 2017). MAGE-A4, a key member of the MAGE-A family, exhibits high expression in various cancer tissues (including esophageal, head and neck, lung, bladder, melanoma, breast, colorectal, and gastric cancers) as well as in testis and placenta, while showing low expression in other normal tissues (Chambost et al. 2000). MAGEA4 represents a potential target for cancer diagnosis and treatment. It participates in the regulation of cell growth, cell cycle, and apoptosis through the expression of p53-related genes, and maintains DNA replication function via translesion synthesis (TLS), playing a crucial role in the development and progression of various malignancies. Additionally, MAGE-A can be processed intracellularly into antigenic peptides, forming complexes with HLA class I molecules. These complexes are then presented to CD8 + T cells via MHC class I molecules, inducing tumor-specific immune responses in cancer patients. Therefore, understanding the functions and mechanisms of MAGEA4 can provide new insights into how to target MAGEA4 for tumor treatment. To facilitate reader comprehension, we have provided a comprehensive table (Table 1) summarizing all immunotherapies investigated in this study.

**Table 1:** Summary of tumor immunotherapy involving MAGE-A4 mentioned in this article

Cancer type	Immunotherapy type	Mechanism	Characteristics	Notes
Lung Cancer	TCR-T	Modifies T cells to express tumor-specific T-cell receptors (TCRs) that identify and attack specific tumor antigens	1. Highly specific for a single antigen; 2.Requires autologous T cell extraction, genetic engineering, and reinfusion; 3. Can recognize intracellular antigens; MHC-restricted	Targets MAGE-A4 epitopes presented on HLA-A2 molecules encoded by HLA-A*02:01 allele
Esophageal Cancer	Tumor Vaccine	Administers tumor-specific peptides to stimulate the patient’s immune system to generate T-cell responses targeting these antigens	1. Elicits immune responses specific to defined peptides; 2. May contain multiple peptides, each with high specificity; 3. Can be personalized or universal; 4. Simple preparation with fewer side effects; 5. May require multiple doses to sustain response due to limited immunogenicity	In a Phase II clinical trial, 24% of patients vaccinated with MAGEA4-derived peptide vaccine developed MAGEA4-specific immune responses
	TCR-T	Same as above	Same as above	Phase I clinical trials demonstrated that MAGEA4-specific TCR-engineered T cells achieved long-term persistence in patients and maintained anti-tumor activity
Breast Cancer	Multi-Antigen Targeted T Cells	Enables T cells to recognize multiple tumor antigens, potentially using combined TCRs or TCRs with CAR constructs	1. Recognizes multiple antigens, including surface and intracellular antigens; 2. Highly individualized with potential for sustained activity in vivo; 3. Targets multiple antigens to reduce risk of tumor escape; 4. Potential for increased side effects	Multi-antigen-targeted T cells showed good tolerance and achieved tumor control in patients with refractory breast cancer
	Tumor Vaccine	Same as above	Same as above	Specific immune response with IgG1 and IgG3 antibody production led to complete remission in a TNBC (Triple-Negative Breast Cancer) patient
Synovial Sarcoma	TCR-T	Same as above	Same as above	Phase I clinical trials achieved a 44% response rate in patients with synovial sarcoma
Colorectal Cancer	Tumor Vaccine	Same as above	Same as above	Patients gained humoral and cellular immune responses

## Mechanisms of MAGEA4 in tumorigenesis (Fig. 2)

### MAGE-A4 binding to TRIM28 promotes tumor development

TRIM28 (also known as KAP1 or TIF1-β) is a crucial member of the E3 RING ubiquitin ligase family, playing a pivotal role in tumorigenesis and progression. As a large multi-domain protein (110 kDa), TRIM28’s amino terminus comprises a RING finger structure, two B-box structures, and a leucine zipper coiled-coil region (CC), collectively referred to as the RBCC or TRIM domain (Friedman et al. 1996; Hatakeyama 2011; Peng et al. 2000a; Peng et al. 2000b). The RING finger domain is essential for its E3 ubiquitin ligase function.

**Fig. 2:**
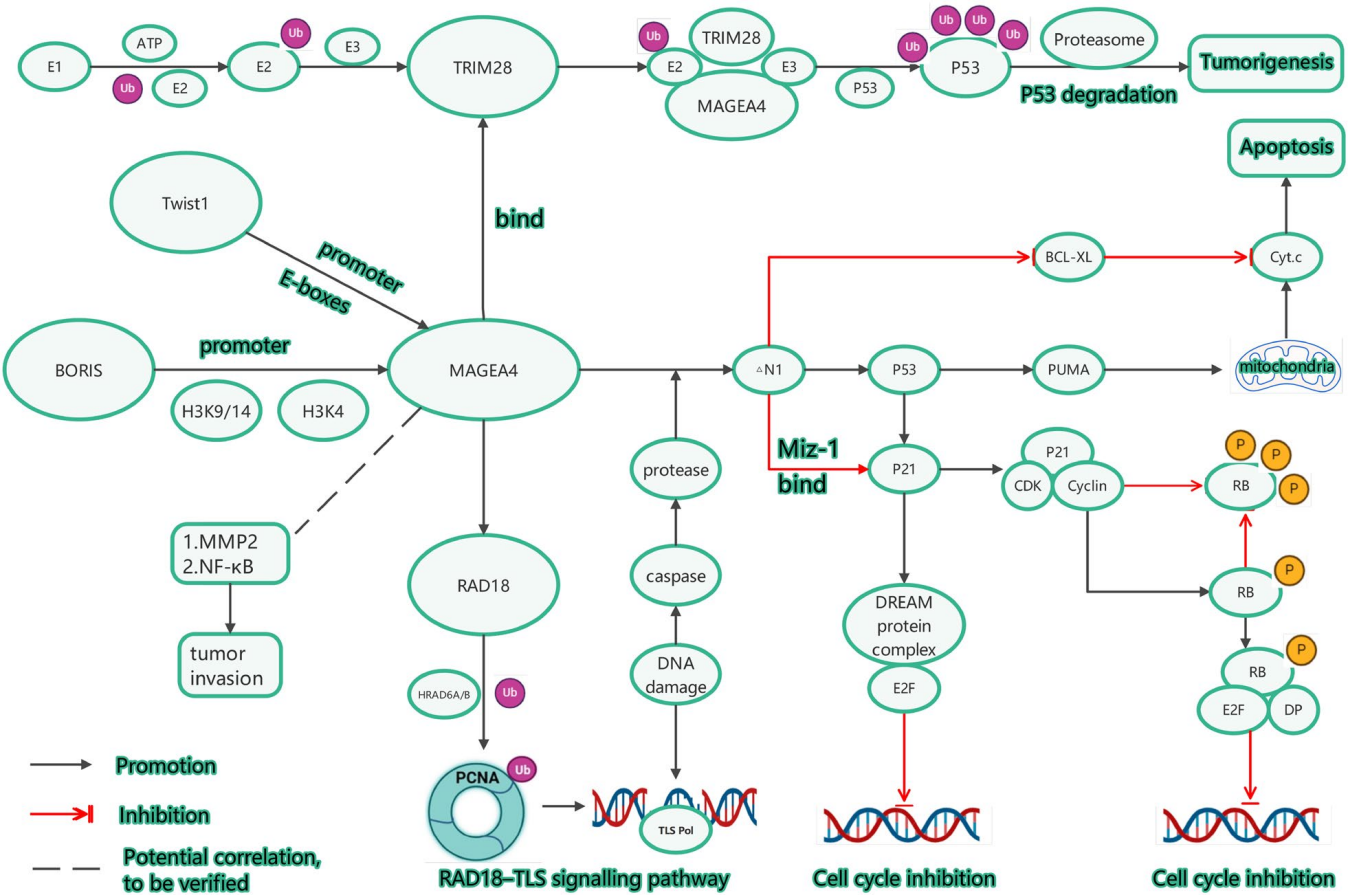
MAGEA4 molecular mechanism diagram (Bhan et al. 2011; Forghanifard et al. 2017; Sakurai et al. 2004; Engeland 2022; Gao et al. 2016; Morocz et al. 2024; Lee et al. 2015; Liu et al. 2014)

In the ubiquitination process, E1 activating enzymes first activate ubiquitin molecules, subsequently transferring them to E2 conjugating enzymes (Dikic and Schulman 2023). The MAGE-A protein family, particularly MAGE-A4, serves as a critical binding partner for TRIM28, significantly enhancing its E3 ubiquitin ligase activity. This enhancement promotes TRIM28's recognition and binding to its substrate protein p53, accelerating the E2 enzyme-mediated transfer of ubiquitin molecules to p53 via its RING domain (Yang et al. 2007; Doyle et al. 2010). This ubiquitination modification targets p53 for degradation by the 26S proteasome (Shmueli and Oren 2004).

As a crucial tumor suppressor, p53 degradation leads to impairment of key defense mechanisms such as cell cycle regulation, DNA repair, and apoptosis, thereby promoting tumor initiation and progression (Eischen 2016; Vogelstein et al. 2000; Vousden and Lane 2007; Braithwaite et al. 2006). TRIM28's multi-domain structure enables it to form complexes with MAGE-A4 and modulate its E3 ubiquitin ligase activity, highlighting the intricate structure–function relationship that plays a critical role in tumorigenesis. This discovery not only elucidates the synergistic mechanism of MAGE-A4 and TRIM28 in tumor development but also provides a theoretical foundation for developing novel anti-cancer strategies targeting this pathway.

### BORIS and TWIST1 as regulatory factors of MAGEA4, promoting MAGEA4 expression

BORIS, a proto-oncogene, is the only known paralogue of CTCF and is also referred to as CTCF-like factor (CTCFL). It shares an almost identical 11-zinc finger domain structure with the transcriptional repressor CTCF. BORIS regulates tumor initiation and progression through various mechanisms, including competitively binding to CTCF's DNA targets and interfering with CTCF function, participating in epigenetic regulation, and promoting the generation of tumor stem cells (Pugacheva et al. 2024). Notably, it acts as a promoter to enhance MAGEA4 expression. Studies have demonstrated that BORIS positively regulates MAGEA4 expression by binding to the MAGEA4 promoter region and inducing a more open chromatin conformation. This process is accompanied by the enrichment of active histone modifications, such as acetylated H3K9/14 and trimethylated H3K4, which are associated with transcriptionally active chromatin structures. Interestingly, while BORIS induction is linked to promoter demethylation in the case of MAGEA3, BORIS's regulatory effect on MAGEA4 appears to be independent of promoter demethylation (Bhan et al. 2011). This differential regulation highlights the complex and gene-specific nature of BORIS-mediated transcriptional control in cancer biology.

TWIST1 is a transcription factor belonging to the basic helix-loop-helix (bHLH) family (Thisse et al. 1987). It plays crucial roles in various biological processes, including embryonic development, cell migration, cell differentiation, and tissue regeneration (Ozdemir et al. 2011; Connerney et al. 2006; Cheng et al. 2007; Lee et al. 2006; Zhang et al. 2008). TWIST1 gene expression has been found to be upregulated in multiple types of cancers, closely associated with tumor invasiveness, metastasis, and poor prognosis (Hosono et al. 2007; Niu et al. 2007; Ohuchida et al. 2007; Shibata et al. 2008). Research has shown that TWIST1, as a transcription factor, can upregulate MAGEA4 expression by indirectly binding to the E-box regions of the MAGEA4 promoter sequence, thereby participating in tumor regulation (Forghanifard et al. 2017).

### 2.3 MAGEA4 promotes apoptosis in DNA damage response by regulating p53 signaling pathway and cell cycle molecules

Under DNA damage conditions, the MAGEA4 protein is cleaved by proteases such as caspases, producing a C-terminal fragment MAGEA4ΔN1 with specialized functions. This fragment coordinates cell cycle inhibition and apoptosis by regulating the p53 signaling pathway and related molecules. MAGEA4ΔN1 primarily enhances p53 stability and activity, thereby increasing its regulatory capacity over downstream target genes (such as PUMA and BCL-XL). Unlike typical p53 activation, MAGEA4ΔN1 also suppresses the transcription and protein levels of p21/CDKN1A, directing cell fate towards apoptosis rather than cell cycle arrest.

Mechanistically, MAGEA4ΔN1 inhibits p21 expression through interaction with the transcription factor Miz-1 (Sakurai et al. 2004). Reduced p21 levels weaken its inhibition of cyclin-dependent kinases (CDKs) and their partner proteins, Cyclins, diminishing cell cycle arrest signals. Moreover, the decrease in p21 indirectly affects the formation of the DREAM complex, subsequently influencing the suppression of cell cycle-related genes by factors such as E2F4, DP, and RB (Engeland 2022). These effects collectively attenuate cell cycle arrest, thereby providing conditions for apoptotic signaling.

In promoting apoptosis, MAGEA4ΔN1 enhances p53-mediated transcriptional activation of the pro-apoptotic gene PUMA while inhibiting the expression of the anti-apoptotic protein BCL-XL. This increases mitochondrial outer membrane permeability, leading to the release of cytochrome c (Cyt.c) into the cytoplasm. The release of Cyt.c activates the caspase cascade, ultimately inducing apoptosis. Notably, MAGEA4ΔN1 can induce apoptosis even in cells lacking functional p53, suggesting the existence of other unknown molecular mechanisms (Sakurai et al. 2004).

In summary, MAGEA4ΔN1 effectively promotes cellular inclination towards apoptosis rather than proliferation in response to DNA damage by regulating p53, p21, the DREAM complex, and other molecules, thereby helping to prevent tumor development. This discovery not only deepens our understanding of cellular stress responses but also provides potential targets for novel anti-cancer strategies.

### MAGEA4 maintains DNA replication through activation of translesion synthesis (TLS) mechanism

MAGEA4, as a binding partner of the E3 ubiquitin ligase RAD18, significantly enhances RAD18’s stability (Gao et al. 2016). RAD18 plays a crucial role in DNA replication and repair processes, especially when dealing with damaged DNA. By promoting the monoubiquitination of proliferating cell nuclear antigen (PCNA), RAD18 activates the Fanconi anemia (FA) pathway and recruits TLS polymerases such as DNA polymerase η (pol η) and DNA polymerase ι (pol ι), enabling cancer cells to continue DNA synthesis in damaged genomes (Morocz et al. 2024).

The binding of MAGEA4 to RAD18 may reprogram ubiquitin signaling, further enhancing the activation of the TLS pathway. However, the activation of TLS is often associated with error-prone tendencies, and MAGEA4's action may lead to the accumulation of mutations in cancer cell genomes, thereby influencing cancer progression. HRAD6, acting as an E2 ubiquitin-conjugating enzyme, works in concert with RAD18 to mark PCNA and promote the recruitment of DNA polymerases, ensuring the smooth progression of DNA replication (Gao et al. 2016).

These findings suggest that MAGEA4 may play a significant role in the genomic maintenance of cancer cells by regulating ubiquitin signaling and DNA damage tolerance mechanisms. This helps explain how cancer cells survive and evolve under constant genetic stress.

Since there are many abbreviations mentioned in the MAGEA4 molecular mechanism diagram, we list all the abbreviations in Table 2.

**Table 2:** Abbreviations

Abbreviation	Full Name
E1	Ubiquitin-activating enzyme E1
Ub	ubiquitin
ATP	Adenosine Triphosphate
E2	Ubiquitin-conjugating enzyme E2
E3	Ubiquitin-protein ligase E3
TRIM28	Tripartite Motif-Containing 28
P53	Tumor protein P53
Twist1	Twist-related protein 1
E-BOX	Enhancer Box
BORIS	Brother of the Regulator of Imprinted Sites
H3K9	Histone H3 Lysine 9
H3K14	Histone H3 Lysine 14
H3K4	Histone H3 Lysine 4
MAGEA4	MAGE family member A4
BCL-XL	B-cell lymphoma-extra large
Cyt.c	Cytochrome c
PUMA	p53 upregulated modulator of apoptosis
MMP2	Matrix Metalloproteinase 2
NF-κB	Nuclear Factor kappa B
RAD18	E3 ubiquitin-protein ligase RAD18
HRAD6A	Human Radiation sensitivity gene 6A
HRAD6B	Human Homolog of Yeast RAD6B
PCNA	Proliferating Cell Nuclear Antigen
TLS pol	Translesion Synthesis Polymerase
P21	Cyclin-Dependent Kinase Inhibitor 1
Miz-1	Myc-interacting zinc finger protein 1
E2F	E2 Transcription Factor
CDK	Cyclin-Dependent Kinase
RB	Retinoblastoma protein
P	Phosphorylation
DP	Dimerization Partner

## The role of MAGEA4 in tumorigenesis

Members of the MAGE-A family are expressed in a wide variety of tumor tissues, including hepatocellular carcinoma, non-small cell lung cancer, bladder cancer, vulvar cancer, colorectal tumors, and salivary gland tumors (Peng et al. 2005; Hou et al. 2020; Bergeron et al. 2009; Bellati et al. 2007; Park et al. 2002, 2012). Furthermore, recent studies have also confirmed the expression of MAGE-A4 in various malignancies such as breast cancer, esophageal squamous cell carcinoma, melanoma, and oral squamous cell carcinoma (Xiao et al. 2023; Sani et al. 2018; Freiberger et al. 2023; Montoro et al. 2012). This widespread distribution suggests that MAGE-A4 may have significant biological functions and clinical implications across multiple tumor types. The extensive presence of MAGE-A4 in diverse cancer types underscores its potential importance as a biomarker for cancer diagnosis and prognosis, as well as a potential therapeutic target. The consistent expression of MAGE-A4 across various malignancies also hints at a possible common mechanism in cancer development or progression, which warrants further investigation to elucidate its precise role in oncogenesis and tumor maintenance.

### The role of MAGEA4 in lung cancer

The expression of MAGE-A4 (Melanoma-Associated Antigen A4) in lung cancer is associated with prognosis. MAGE-A4, a cancer/testis antigen, is overexpressed in various malignancies, including non-small cell lung cancer (NSCLC) (Hou et al. 2020). Studies have shown that MAGE-A4 is expressed in 4 out of 8 NSCLC cell lines, with an expression rate of 25.4% in clinical lung cancer specimens. Notably, the prognostic value of MAGE-A4 depends on its intracellular localization and p53 status. Patients with nuclear MAGE-A4 expression but lacking p53 expression had significantly lower survival rates compared to those expressing both nuclear MAGE-A4 and p53. In fact, multivariate analysis identified nuclear MAGE-A4 as an independent prognostic factor (P = 0.0042), albeit only in the absence of p53 (Fujiwara-Kuroda et al. 2018).

Further research has demonstrated that MAGE-A4 expression in peripheral blood of lung cancer patients correlates with clinical staging, tumor size, lymph node metastasis, and distant metastasis. MAGE-A4 expression is associated with lower overall survival in lung cancer patients (Gu et al. 2018). MAGEA4 protein expression is related to immune cell infiltration, with high MAGEA4 expression associated with M2-type macrophages (CD163) and regulatory T cells (FOXP3), while low MAGEA4 expression is associated with T cells (CD3), suggesting MAGEA4 may have immunogenic effects in the local tumor microenvironment (Hikmet et al. 2023).

In NSCLC, MAGE-A4 has been reported as a target for T cell-specific immunotherapy. Kathrin Davari, Tristan Holland, et al. employed a TCR-T approach, processing MAGE-A4 epitopes presented on HLA-A2 molecules encoded by the HLA-A*02:01 allele, enabling CD4 T cells to kill MAGE-A4 positive tumor cells (Davari, et al. 2021). Normal expression of HLA class-I in NSCLC is associated with good prognosis in MAGEA4-positive patients, while downregulation of HLA class-I may lead to disease progression, with smoking being one factor contributing to HLA class-I downregulation (Hanagiri et al. 2013).

Research indicates that high MAGE-A4 expression in lung adenocarcinoma patients is associated with response to immune checkpoint inhibitor (ICI) therapy. Leana Rich M Herrera, using “reverse vaccinology” and “immunoinformatics” approaches, computationally predicted a multi-epitope vaccine targeting MAGEA4 expression in NSCLC. The immunogenicity of the epitopes included in the vaccine was validated, and their antigenicity, non-allergenicity, non-toxicity, and physicochemical stability were assessed, highlighting the close relationship between MAGEA4 and immunotherapy (Herrera 2021). Additionally, studies have shown that Melanoma Antigen A4 is expressed in NSCLC and promotes cell apoptosis (Peikert et al. 2006).

In conclusion, MAGE-A4 likely plays a crucial role in the occurrence and development of lung cancer, making it an important target for lung cancer treatment.

### The role of MAGEA4 in liver cancer

MAGE-A4, a tumor-associated antigen, exhibits an mRNA expression rate of up to 33.8% in hepatocellular carcinoma (HCC) tissues. Studies have revealed a positive correlation between MAGE-A4 expression levels and elevated serum AFP, advanced tumor stage, and high proliferation marker Ki-67, suggesting a close association with HCC's malignant phenotype and progression (Wang et al. 2015). Significant differences in MAGE-A4 positive expression rates are observed in peripheral blood mononuclear cells of HCC patients at different pathological stages, with rates reaching 40% in advanced patients compared to only 6.7% in early stages, indicating a positive correlation between MAGE-A4 expression levels and disease progression. Notably, MAGE-A4 is virtually unexpressed in healthy populations and chronic liver disease patients, suggesting its specificity in HCC (Hussein et al. 2012). These findings collectively suggest that MAGE-A4 expression could serve as a potential molecular marker for predicting HCC recurrence, metastasis, and prognosis assessment.

Functional studies have shown that the C-terminal fragment of MAGE-A4 protein (MAGE-A4N1) can induce tumor cell apoptosis. The molecular mechanism involves binding to the Miz-1 protein, inhibiting p21Cip1 transcription, thereby promoting the apoptotic process. The MAGE-A4N1 fusion protein can inhibit the anchorage-independent growth of HCC cells, demonstrating potential anti-cancer activity (Sakurai et al. 2004). Furthermore, MAGE-A4 can also bind to the Gankyrin protein, partially inhibiting Gankyrin overexpression-induced anchorage-independent growth and nude mouse tumor formation, revealing a new mechanism of MAGE-A4 in regulating tumor activity (Nagao et al. 2003).

Due to the highly restricted expression of MAGE-A4 in normal tissues, its abnormal expression in HCC provides an ideal target for tumor immunotherapy. Simultaneously, the detection of the MAGE-A4 gene in blood, especially in follow-up monitoring, may aid in assessing HCC prognosis and monitoring treatment response (Hussein et al. 2012). The interaction between MAGE-A4 and the chemotherapy drug doxorubicin also brings new insights into liver cancer treatment. Research has found that doxorubicin can promote MAGE-A4 cleavage by activating the proteasome, producing C-terminal fragments with pro-apoptotic activity, thereby enhancing the sensitivity of liver cancer cells to doxorubicin. This discovery not only helps understand the mechanism of doxorubicin but also provides the possibility of developing MAGE-A4’s C-terminal fragment as a novel anti-cancer drug (Sakurai et al. 2011).

In conclusion, the abnormal expression of MAGE-A4 in HCC and the unique functions of its encoded protein demonstrate potential clinical application value in disease diagnosis, prognosis assessment, and targeted therapy. In-depth research on the molecular regulatory network of MAGE-A4 and its role in HCC occurrence and development will lay the theoretical foundation for developing new diagnostic and therapeutic strategies. The multifaceted involvement of MAGE-A4 in HCC biology underscores its significance as a research focus in hepatocellular carcinoma.

### The role of MAGEA4 in esophageal cancer

Esophageal cancer is a common and highly invasive malignant tumor of the digestive system, with esophageal squamous cell carcinoma (ESCC) being the predominant pathological type. Due to the lack of effective early screening and diagnostic methods, most ESCC patients are diagnosed at an advanced stage, resulting in poor prognosis (Mousavi et al. 2009). Therefore, there is an urgent need to discover new biomarkers for early diagnosis and personalized treatment of ESCC. MAGE-A4, a tumor-associated antigen, has been shown in multiple studies to be highly expressed in ESCC tissues (Sani et al. 2018; Tang et al. 2016). Real-time RT-PCR and immunohistochemical analyses have demonstrated significant mRNA and protein expression of MAGE-A4 in 60% to 90% of ESCC samples (Cuffel et al. 2011; Bujas et al. 2011). Notably, MAGE-A4 expression exhibits heterogeneity within tumors, with some regions showing higher expression intensity.

Importantly, MAGE-A4 expression levels are significantly correlated with tumor invasion depth, clinical stage, and lymph node metastasis status, suggesting its potential involvement in ESCC progression and metastasis (Sani et al. 2018). Multiple cohort studies have found that high MAGE-A4 expression levels in ESCC tissues are significantly associated with poor patient prognosis. MAGE-A4-positive patients have markedly shorter overall survival compared to negative patients. Even in early-stage ESCC patients, high MAGE-A4 expression is an independent indicator of poor prognosis (Tang et al. 2016). These results suggest that MAGE-A4 can serve as a molecular marker for predicting ESCC patient prognosis.

Functional studies further indicate that MAGE-A4 may play a carcinogenic driving role in the occurrence and development of ESCC through various molecular mechanisms. At the molecular level, MAGEA4 may play a role in tumor progression by modulating the NF-κB and MMP2 signaling pathways (Liu et al. 2014). On the other hand, the TWIST1 transcription factor can bind to the MAGE-A4 promoter region and upregulate its expression (Forghanifard et al. 2017). Therefore, MAGE-A4 is considered a potential oncogene in ESCC.

**Fig. 3:**
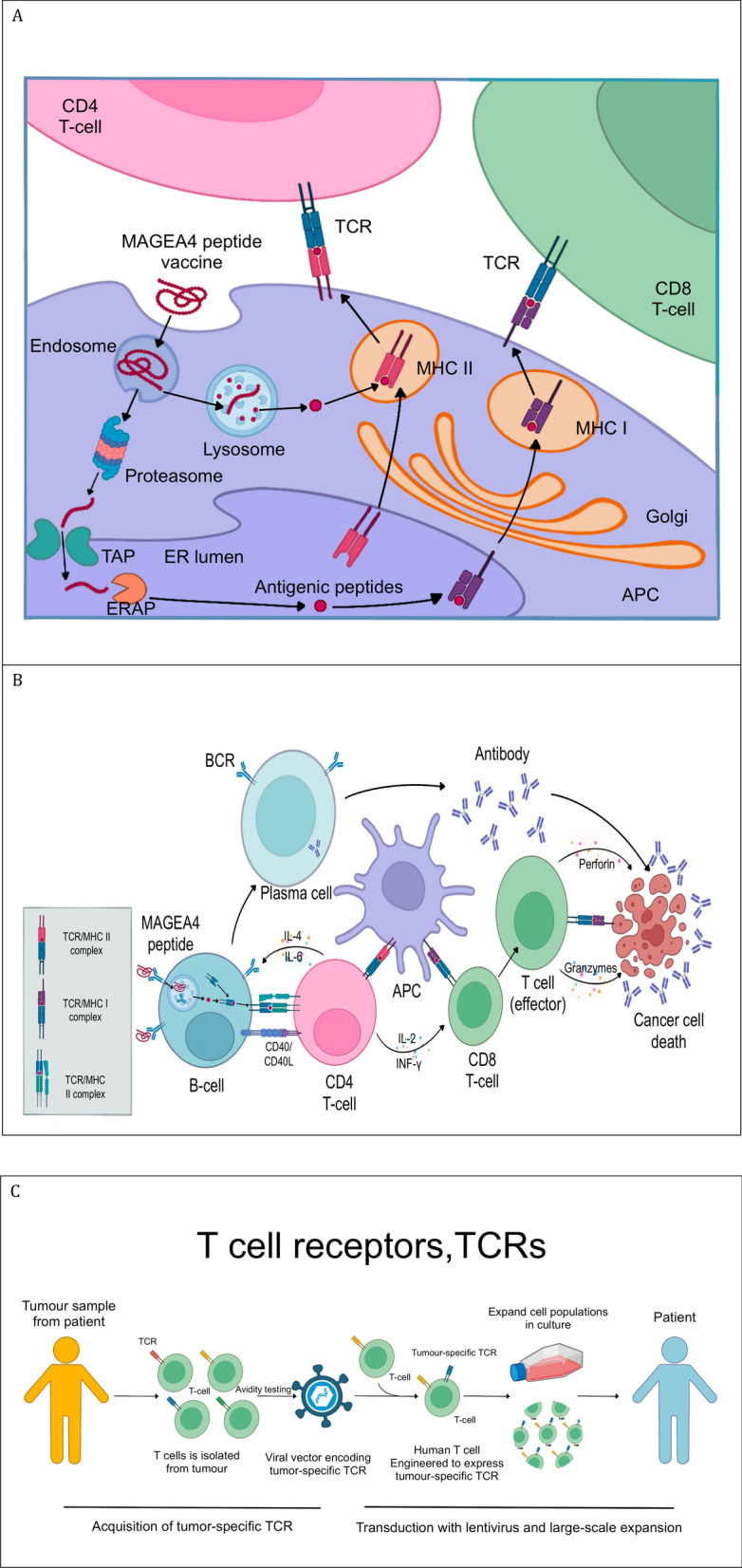
MAGEA4 in Tumor Immunotherapy. **A** Antigen Presentation of MAGEA4 Peptide Vaccines (Targeting the cancer mutanome 2017), **B** Mechanisms of Action of MAGEA4 Peptide Vaccines (Alsalloum et al. 2023), **C** TCR-T Therapy Targeting MAGEA4 (Restifo et al. 2012)

Immunologically, MAGE-A4 can induce autoantibodies and T cell immune responses in esophageal cancer patients. Some non-vaccinated esophageal cancer patients naturally acquire humoral immune responses to MAGE-A4 and MAGE-A4-specific CD4 + and CD8 + T cell responses. This lays the foundation for developing active immunotherapy targeting MAGE-A4 (Saito et al. 2014). Multiple clinical trials have evaluated the safety and immunogenicity of MAGE-A4 protein vaccines in patients with solid tumors, including esophageal cancer. Results show that MAGE-A4 protein vaccines have good safety profiles and can induce or enhance MAGE-A4-specific antibody responses in patients (Saito et al. 2014). In a phase II clinical trial, 24% of patients vaccinated with MAGE-A4 developed MAGE-A4-specific immune responses (Ueda et al. 2018). (As shown in Fig. 3A and B).

Another immunotherapy strategy is MAGE-A4-specific TCR-transduced T cell therapy (As shown in Fig. 3C). Researchers collect peripheral blood lymphocytes from patients, transduce these cells with T cell receptor (TCR) genes that recognize MAGE-A4, enabling them to acquire specific killing ability against MAGE-A4. After in vitro expansion, these TCR-engineered T cells are reinfused into patients (Tanaka et al. 2017). A phase I clinical trial for recurrent esophageal cancer patients showed that MAGE-A4-specific TCR-transduced T cells could survive long-term in patients and maintain reactivity against tumors, potentially benefiting patients with lower tumor burden. Although no significant tumor shrinkage was observed in the short term, this strategy demonstrated potential clinical value (Kageyama et al. 2015).

Studies have found that patients with high co-expression of MAGE-A4 and MHC class I molecules in tumor cells are more likely to develop MAGE-A4-specific immune responses after MAGE-A4 vaccination and have longer overall survival. Therefore, co-expression of MAGE-A4 and MHC molecules in tumor cells may be an important prognostic marker for the efficacy of MAGE-A4 immunotherapy (Saito et al. 2014). NY-ESO-1 is another tumor-associated antigen co-expressed with MAGE-A4 in some esophageal cancers. Research has shown that patients co-expressing these two antigens have poorer prognosis, but may experience antigen spread to NY-ESO-1 after MAGE-A4 vaccination, potentially leading to better treatment outcomes (Bujas et al. 2011; Ueda et al. 2018; Chen et al. 2017). Thus, NY-ESO-1 expression status is also an important factor in evaluating the potential of MAGE-A4 immunotherapy.

In addition to the above factors, immunosuppressive elements in the local tumor microenvironment, such as regulatory T cells, may also affect the efficacy of MAGE-A4 immunotherapy (Kawada et al. 2012). Overall, MAGE-A4, as a tumor-associated antigen, demonstrates good immunogenicity in esophageal cancer patients. Various immunotherapy strategies based on MAGE-A4 are under clinical investigation and show some clinical benefits (Table 3). However, the factors affecting immunotherapy efficacy are complex, requiring further research to optimize treatment strategies for greater benefits to esophageal cancer patients.

**Table 3:** Clinical Trial for MAGE-A4 Positive Patients (Data source: ClinicalTrials.gov, URL: https://clinicaltrials.gov/search?term=magea4&viewType=Table)

NCT number	Study title	Study STATUS	Conditions
NCT06170294	MAGE-A4-directed TCR-T in the Treatment Amongst Subjects With Advanced Solid Tumors	Recruiting	Advanced Solid Tumor
NCT03247309	TCR-engineered T Cells in Solid Tumors (ACTengine IMA201-101)	Completed	Solid Tumor|Cancer|Recurrent Solid Tumors|Refractory Solid Tumors
NCT01694472	Clinical Safety and Preliminary Efficacy of MAGE-A4 TCR Gene-Modified T Cells to Treat Malignant Tumors	Unknown	Malignant Solid Tumors
NCT06402201	First in Human Study of CDR404 in HLA-A*02:01 Participants With MAGE-A4 Expressing Solid Tumors	Recruiting	Select Advanced Solid Tumors
NCT02096614	Investigator Initiated Phase 1 Study of TBI-1201	Completed	Solid Tumors
NCT04752358	ADP-A2M4CD8 in HLA-A2 + Subjects With MAGE-A4 Positive Esophageal or Esophagogastric Junction Cancers (SURPASS-2)	Terminated	Esophageal Cancer|Esophagogastric Junction Cancer
NCT06372574	A Study of RO7617991 in Patients With Locally Advanced or Metastatic MAGE-A4-Positive Solid Tumors	Withdrawn	Refractory Cancer|Recurrent Cancer|Solid Tumor, Adult
NCT03973333	Safety and Efficacy of IMC-C103C as Monotherapy and in Combination With Atezolizumab	Terminated	Select advanced solid tumors
NCT04044859	ADP-A2M4CD8 as Monotherapy or in Combination With Either Nivolumab or Pembrolizumab in HLA-A2 + Subjects With MAGE-A4 Positive Tumors (SURPASS)	Recruiting	Endometrial Cancer|Esophageal Cancer|Esophagogastric Junction (EGJ)|Gastric (Stomach) Cancer|Head and Neck Cancer|Melanoma|Ovarian Cancer|Non-small Cell Lung (NSCLC)|Urothelial Cancer
NCT05642455	SPEARHEAD-3 Pediatric Study	Recruiting	Synovial Sarcoma|Malignant Peripheral Nerve Sheath Tumor (MPNST)|Neuroblastoma|Osteosarcoma
NCT05601752	ADP-A2M4CD8 Monotherapy and in Combination With Nivolumab in HLA-A2 + Subjects With MAGE-A4 Positive Ovarian Cancer (SURPASS-3)	Recruiting	Ovarian Cancer
NCT05129280	A Study to Evaluate Safety, Pharmacokinetics, and Preliminary Anti-tumor Activity of RO7444973 in Participants With Unresectable and/or Metastatic MAGE-A4-positive Solid Tumors	Terminated	Solid Tumors
NCT04044768	Spearhead 1 Study in Subjects With Advanced Synovial Sarcoma or Myxoid/Round Cell Liposarcoma	Recruiting	Synovial Sarcoma|Myxoid Liposarcoma
NCT03132922	MAGE-A4^c1°32^ T for Multi-Tumor	Active_not_recruiting	Urinary Bladder Cancer|Melanoma|Head and Neck Cancer|Ovarian Cancer|Non-Small Cell Lung Cancer|Esophageal Cancer|Gastric Cancer|Synovial Sarcoma|Myxoid Round Cell Liposarcoma|Gastroesophageal Junction
NCT04408898	SPEARHEAD 2 Study in Subjects With Recurrent or Metastatic Head and Neck Cancer	Withdrawn	Head and Neck Cancer
NCT04199559	Evaluating Combination Therapy Using Autologous Dendritic Cells Pulsed With Antigen Peptides and Nivolumab for Subjects With Advanced Non-Small Cell Lung Cancer	Unknown	NSCLC
NCT02636855	Screening Protocol for Tumor Antigen Expression Profiling and HLA Typing for Eligibility Determination	Recruiting	Solid and Hematological Malignancies
NCT02239861	TAA-Specific CTLS for Solid Tumors (TACTASOM)	Completed	Rhabdomyosarcoma
NCT03356808	Antigen-specific T Cells Against Lung Cancer	Unknown	Lung Cancer
NCT03093350	TACTIC—TAA Specific Cytotoxic T Lymphocytes in Patients With Breast Cancer	Active_not_recruiting	Breast Cancer
NCT03970746	Safety, Immunogenicity and Preliminary Clinical Activity Study of PDC*lung01 Cancer Vaccine in NSCLC	Active_not_recruiting	Non Small Cell Lung Cancer
NCT03192462	TAA Specific Cytotoxic T Lymphocytes in Patients With Pancreatic Cancer	Active_not_recruiting	Pancreatic Cancer
NCT02291848	Tumor-Associated Antigen-Specific Cytotoxic T-Lymphocytes for Multiple Myeloma	Recruiting	Multiple Myeloma
NCT05572944	Formatting the Risk Prediction Models for Never-Smoking Lung Cancer	Recruiting	Lung Cancer
NCT01333046	Administration of TAA-Specific CTLs; Hodgkin or Non-Hodgkin Lymphoma; TACTAL	ACTIVE_NOT_RECRUITING	Hodgkin Lymphoma|Non-Hodgkin Lymphoma|Hodgkin Disease
NCT05134740	(TAA)-Specific Cytotoxic T-Lymphocytes to Pediatric Patients With Lymphomas (pediTACTAL)	Recruiting	Hodgkins Lymphoma|Non Hodgkins Lymphoma

At the basic research level, researchers have identified the HLA-A2-restricted epitope p286-1Y2L9L derived from MAGE-A4, which can induce specific cytotoxic T lymphocytes (CTLs) capable of killing MAGE-A4-positive tumor cells. In HLA-A2.1/Kb transgenic mouse models, CTLs induced by the p286-1Y2L9L peptide can recognize and kill MAGE-A4-positive tumor cells (Wu et al. 2011). In NOG mice (SCID/γcnull mice), MAGE-A4-specific TCR-transduced human PBMCs can infiltrate tumor tissues and inhibit tumor growth (Shirakura et al. 2012). These basic studies have laid the foundation for the clinical translation of MAGE-A4 immunotherapy.

### The role of MAGEA4 in head and neck cancers

MAGE-A4 is a tumor-associated antigen that exhibits elevated expression levels in various head and neck tumors. Studies have revealed that MAGE-A4 is expressed in approximately 50% of cases of nasopharyngeal carcinoma, oral squamous cell carcinoma, and laryngeal cancer, with expression rates reaching up to 90% in certain cancer types (Figueiredo et al. 2006; Brisam et al. 2016). Notably, MAGE-A4 expression within tumor tissues is heterogeneous, with some regions displaying higher expression levels. In contrast, it is virtually absent in normal tissues, with only minimal expression in specialized tissues such as the testes, thus conferring high tumor specificity (Figueiredo et al. 2011).Multiple studies have demonstrated that high MAGE-A4 expression levels correlate with advanced clinical stages, increased metastatic potential (e.g., lymph node metastasis), and poorer prognosis, including shortened overall survival. MAGE-A4 expression is more prevalent in advanced, high-grade head and neck tumors (Brisam et al. 2016). Research has directly confirmed MAGE-A4 as an independent prognostic factor for adverse outcomes (Laban et al. 2019).

At the molecular level, MAGE-A4 may promote tumor cell proliferation by suppressing the expression of apoptosis-related genes (such as p53 downstream genes BAX and CDKN1A). Its expression is also associated with DNA methylation levels, and demethylating agents can induce upregulation of MAGE-A4 expression (Bhan et al. 2012).

Due to its high specificity and overexpression in tumor tissues, MAGE-A4 is considered a potential tumor-associated antigen and a target for cancer immunotherapy. Studies have isolated MAGE-A4-reactive CD4 + T cells from unvaccinated head and neck cancer patients and identified novel helper epitopes of MAGE-A4, laying the foundation for vaccine development. Combining MAGE-A4 with other MAGE family members (e.g., MAGE-A3) or other tumor-associated antigens may lead to more effective multi-target immunotherapy strategies (Cesson et al. 2011).Furthermore, detection of MAGE-A4 expression may aid in the early detection and diagnosis of head and neck tumors. In some cases, its expression may reveal potential risks of malignant transformation, guiding timely treatment and improving diagnostic accuracy as a molecular biomarker. Research has shown that MAGE-A4 expression positively correlates with the expression of other cancer-testis antigens such as LAGE1 and NY-ESO1, suggesting these antigens may play synergistic roles in tumor development and progression (Forghanifard et al. 2011).

The expression pattern of MAGE-A4 in head and neck tumors may also be associated with clinical factors such as patients' smoking habits (Figueiredo et al. 2006). Additionally, MAGE-A4 expression varies across different tumor regions, often exhibiting lower expression in invasive front areas (Brisam et al. 2016).

In conclusion, MAGE-A4 demonstrates significant biological and clinical value in head and neck tumors. It not only serves as a prognostic marker but, more importantly, holds potential applications in cancer immunotherapy and early diagnosis. These aspects warrant further in-depth research and development for clinical utilization.

### The role of MAGEA4 in breast cancer

The aberrant expression of cancer/testis antigen (CTA) MAGE-A4 in triple-negative breast cancer (TNBC) and certain other breast cancer subtypes, along with its clinical significance, has garnered considerable attention. Studies have revealed MAGE-A4 expression in 29.41% of TNBC samples, with its positive expression associated with prolonged disease-free survival, suggesting its potential as an immunotherapeutic target for TNBC. At least one of MAGE-A4, NY-ESO-1, or PRAME is expressed in 76.47% of TNBC cases. MAGE-A4 expression correlates with WHO tumor grade, indicating lower tumor differentiation, but shows no significant association with other clinical factors such as age, lymph node status, tumor size, lymphovascular invasion, or perineural invasion (Xiao et al. 2023).

Artificial intelligence models have identified that high MAGE-A4 expression in breast cancer is associated with reduced chemotherapy sensitivity. Its downregulation can inhibit cell migration and wound healing, potentially relating to tumor metastatic potential. Mechanistic studies indicate that MAGE-A4 may contribute to chemotherapy resistance acquisition through the CDKN2A/STAT3 pathway, and is associated with the expression of PD-L1 and the apoptosis-related protein caspase-3, influencing tumor cell survival and apoptosis processes (Wan et al. 2023).

Multiple preclinical studies have evaluated immunotherapeutic strategies targeting MAGE-A4. Multi-antigen targeted T cells capable of recognizing MAGE-A4 and eliciting immune responses have shown some disease control in a subset of patients (Hoyos et al. 2022). Another approach involves developing high-affinity, co-receptor-independent MAGE-A4-specific TCR-T cell products. Preclinical evaluations demonstrate their ability to activate various T cell subsets, potentially offering enhanced cellular responses in clinical settings (Davari, et al. 2021).Furthermore, cancer vaccines targeting MAGE-A4 or MAGE-A family members, such as the H/K-HELP vaccine, have shown promising efficacy in some patients by stimulating Th1 and Tc1 cell cancer-specific immune responses and inducing IgG1 and IgG3 antibody production. Notably, one TNBC patient achieved complete remission (Nishimura 2012).

MAGE-A4 is also highly expressed in BRCA1/2-associated breast cancers, with 13 out of 26 cases (50%) testing positive. Given its absence in normal breast tissue, MAGE-A4 emerges as a potential target for developing preventive vaccines for BRCA mutation carriers (Adams et al. 2011).

Immune-enriched TNBC presents a distinct immune microenvironment and therapeutic targets compared to ER-positive breast cancer, with overexpression of immune-related genes such as IFNG, PD-L1, and CTLA4 in TNBC, necessitating different immunotherapeutic strategies (O'Meara, et al. 2020).

MAGE-A4 expression is significant in TNBC and HER2-positive/ER-negative breast cancers, detectable in tumor interstitial fluid and serum. Its positive expression correlates with high-grade tumor classification and may serve as a prognostic indicator for breast cancer (Cabezon et al. 2013; Hussein et al. 2011).

As a member of the MAGE-A family, MAGE-A4 may regulate germ cell proliferation, differentiation, and survival, with similar functions in cancer cells, involving processes of cell proliferation, stem cell function, and tumor development (Cabezon et al. 2013).

In conclusion, MAGE-A4 represents a potential therapeutic target and prognostic marker for TNBC and certain other breast cancer subtypes, holding significant clinical relevance and research value in the field of tumor immunotherapy.

### The role of MAGEA4 in other cancers

MAGE-A4 is a cancer/testis antigen (CTA) with strictly limited expression in normal tissues but widespread and high expression in various solid tumors and hematological malignancies, playing a significant role in cancer biology. In tenosynovial giant cell tumors, the average rate of MAGE-A4-positive cells reaches 68.9%, showing a significant negative correlation with β-catenin expression (r = −0.64). This suggests that MAGE-A4 may interact with β-catenin, potentially contributing to tumor development (Hashimoto, et al. 2023).

In malignant soft tissue sarcomas, MAGE-A4 positivity strongly correlates with tumor metabolic activity as measured by SUVmax, reflecting its possible influence on tumor biological behavior through regulation of related pathways (Hashimoto et al. 2022). As a CTA, MAGE-A4 plays a role in the immune response to sarcomas, although not all patients respond to it, possibly due to MAGE-A4-mediated tumor immune evasion mechanisms.

A phase I clinical trial using the MAGE-A4-specific TCR-T cell product afamitresgene autoleucel for solid tumor treatment achieved a 44% response rate in synovial sarcoma patients, demonstrating MAGE-A4’s potential in cancer immunotherapy (Hong et al. 2023). In head and neck mucosal melanoma, MAGE-A4 expression reaches 61%, suggesting the potential applicability of MAGE-A4-based immunotherapy strategies, such as MAGE-A4-specific TCR-T cell therapy (Prasad et al. 2004).

MAGE-A4 is closely associated with normal germ cell development. It is expressed in germ cell neoplasia in situ (GCNIS) cells, precursors of testicular germ cell tumors. Through interactions with molecules like Gankyrin and mediation of signaling pathways such as p53, MAGE-A4 may exert anti-proliferative effects and participate in the invasive transformation of GCNIS (Camacho-Moll et al. 2019).

In pancreatic cancer, MAGE-A4 expression is relatively low but may be regulated by epigenetic mechanisms such as DNA methylation and histone deacetylase inhibitors. Due to its absence in normal tissues, MAGE-A4 remains a potential immunotherapeutic target for pancreatic cancer, with strategies like MAGE-A4 peptide vaccines under development (Bert et al. 2002).

MAGE-A4 expression frequency in colorectal cancer is approximately 15%. Although relatively low, it can induce specific T cell responses in some patients, making it a potential T cell vaccine target. Immunohistochemical analysis reveals heterogeneous MAGE-A4 expression in colorectal cancer cells (Alves et al. 2007). Studies have evaluated the ability of MAGE-A4 peptide vaccines, in combination with other adjuvants, to induce specific humoral and cellular immune responses in colorectal cancer patients (Miyauchi et al. 2016; Takahashi et al. 2012).

In bladder cancer, MAGE-A4 expression closely correlates with tumor stage, grade, invasiveness, and prognosis (Patard et al. 1995). MAGE-A4-positive non-muscle-invasive bladder cancer patients show a significantly increased risk of progression to muscle-invasive disease (hazard ratio 3.7) (Bergeron et al. 2009).

In conclusion, MAGE-A4 is extensively involved in the development and progression of various solid tumors and hematological malignancies. Through interactions with multiple molecules and signaling pathways, it plays a crucial role in regulating tumor biological behavior and influences prognosis in certain cancers. Due to its tumor-specific expression, MAGE-A4 represents an ideal tumor-associated antigen for developing novel cancer immunotherapy strategies, such as peptide vaccines and TCR-T cell therapies, showing broad clinical potential.

## Discussion and future directions

This review summarizes the expression characteristics and biological functions of MAGEA4 in various tumors, as well as recent research progress on its potential as a diagnostic marker and therapeutic target. As a crucial member of the cancer/testis antigen family, MAGEA4 exhibits high expression in multiple malignancies while maintaining strictly limited expression in normal tissues. This specific expression pattern renders MAGEA4 an attractive target for tumor diagnosis and treatment.

Extensive research has demonstrated that MAGEA4 plays a crucial role in tumorigenesis and cancer progression. MAGEA4 contributes to tumor regulation through multiple mechanisms, encompassing the ubiquitination and subsequent degradation of the tumor suppressor p53, modulation of cell cycle progression and apoptosis, and alteration of DNA damage repair processes. Specifically, MAGE-A4 forms a complex with TRIM28, augmenting E3 ubiquitin ligase activity, which results in the ubiquitination of the tumor suppressor p53 and its subsequent proteasomal degradation, thus promoting tumorigenesis. Consequently, the development of small molecule inhibitors targeting the MAGE-A4-TRIM28 interaction site to mitigate excessive p53 degradation and subsequently suppress tumor development represents a promising therapeutic strategy (Alsalloum et al. 2023).MAGEA4 exhibits elevated expression in various malignant neoplasms, significantly correlating with poor patient prognosis. Studies indicate that the upregulation of its transcriptional regulators, BORIS and TWIST1, further enhances MAGEA4 expression. Therefore, targeted inhibition of BORIS and TWIST1 may offer a potential therapeutic approach for controlling MAGEA4 expression levels and associated tumor progression.The C-terminal fragment of MAGEA4 (MAGEA4ΔN1) has been shown to promote apoptosis by modulating the p53 signaling pathway and cell cycle molecules, providing a theoretical foundation for developing MAGEA4-related anti-cancer strategies. Intriguingly, MAGEA4ΔN1 simultaneously enhances p53 expression while suppressing p21 levels, revealing the flexibility of p53 in regulating cell fate. Typically, p53 induces cell cycle arrest by upregulating p21, allowing time for repair, while p21’s anti-apoptotic function supports cell survival under mild damage conditions (Engeland 2022; Martinez et al. 2002; He et al. 2020; Yosef et al. 2017). However, MAGEA4ΔN1 inhibits p21, shifting p53 regulation towards the activation of pro-apoptotic genes, thereby accelerating the elimination of severely damaged cells. Thus, MAGEA4ΔN1 expedites apoptosis in cells facing irreversible damage by attenuating p21’s anti-apoptotic effects. This mechanism elucidates MAGEA4ΔN1’s unique role in regulating cellular apoptosis, further expanding the theoretical basis for MAGEA4-related anti-tumor strategies.MAGEA4, as a binding partner of RAD18, enhances its stability and activity in the Translesion Synthesis (TLS) pathway by promoting PCNA ubiquitination to recruit low-fidelity TLS polymerases (such as DNA polymerase η). This interaction enables tumor cells to complete replication in the presence of damaged DNA. Through this regulatory mechanism, MAGEA4 plays a vital role in maintaining the genome of tumor cells and their tolerance to DNA damage, allowing them to survive and evolve under persistent genetic stress. However, due to the low fidelity of TLS polymerases, this pathway is associated with a higher risk of mutations, potentially leading to genomic instability and providing a molecular basis for tumor adaptation and progression (Pande et al. 2015; Lv et al. 2013). These findings not only deepen our understanding of MAGEA4's role in tumor biology but also offer multiple potential targets for developing therapeutic strategies targeting MAGEA4.

Multiple studies indicate that MAGEA4 expression levels closely correlate with clinical staging and prognosis in various tumors. For instance, high MAGEA4 expression is often associated with advanced tumor stage and poorer prognosis in non-small cell lung cancer, esophageal cancer, and bladder cancer, among others. These findings highlight the value of MAGEA4 as a potential diagnostic and prognostic marker. However, due to the heterogeneity of MAGEA4 expression and its variations across different tumor types, its application as a biomarker requires validation through larger-scale, more standardized clinical studies.

MAGEA4, a prominent member of the cancer-testis antigen (CTA) family, represents an ideal target for the development of novel tumor immunotherapy strategies. Its distinctive expression pattern—characterized by high expression in testicular germ cells and absence in most normal adult tissues—coupled with the immune-privileged nature of the testes, renders MAGEA4 a highly promising tumor-specific antigen. The immune-privileged characteristics of the testes encompass the absence of MHC I expression in germ cells, the presence of the blood-testis barrier, and central and peripheral tolerance mechanisms. These factors collectively prevent the presentation of MAGEA4 to T cells under normal physiological conditions, thereby averting autoimmune reactions (Dutta et al. 2022).

MAGEA4 shares several key features with other CTAs, including aberrant activation in various tumors, epigenetic regulation (particularly influenced by DNA methylation), and potential immunogenicity. Furthermore, MAGEA4 exhibits structural and functional similarities with other MAGE-A subfamily members, such as involvement in cell cycle regulation and cell survival. These commonalities provide a theoretical basis for postulating that MAGEA4 may employ immune evasion mechanisms analogous to other CTAs (Lian et al. 2018; Wu et al. 2020).

Theoretically, MAGEA4 expression in tissues outside the testes should elicit a robust immune response, particularly against MAGEA4-expressing tumor cells. However, clinical observations reveal a paradoxical association between high MAGEA4 expression in tumors and poor prognosis, suggesting that MAGEA4-positive cancer cells may employ sophisticated immune evasion mechanisms. Primarily, MAGE-A4 is intracellularly localized, precluding direct recognition by specific antibodies or active immune cells (Bujas et al. 2011). Only a small fraction of intracellular MAGE-A4 is presented on the tumor cell surface via MHC class I proteins (MHC-I), insufficient to induce a potent immune response. Additionally, tumor cells can downregulate MHC-I expression to reduce antigen presentation (Saito et al. 2014).

Based on current immunological knowledge and correlative studies on immune evasion by CTA family members (Daiko et al. 2020; Raza et al. 2020; Wang et al. 2018; Thomas et al. 2018; Helmink et al. 2020), we hypothesize that MAGEA4’s immune evasion strategies may include: enhanced anaerobic glycolysis in tumor cells, depleting glucose and creating a low-ATP environment that impairs immune cell cytotoxicity; lactate production from glycolysis, generating an acidic microenvironment that suppresses anti-tumor immune activity; recruitment of immunosuppressive cells such as regulatory T cells and myeloid-derived suppressor cells; secretion of immunosuppressive cytokines, including TGF-β; induction of T cell exhaustion; and exploitation of the tumor microenvironment's lack of co-stimulatory molecules to induce T cell tolerance. It is crucial to emphasize that these proposed mechanisms require rigorous experimental validation in the context of MAGEA4-positive tumors.

Therapeutic strategies targeting MAGEA4 include peptide vaccines, TCR-T cell therapy, and combination immunotherapies. MAGEA4-targeted vaccines have yielded encouraging results in inducing humoral and cellular immune responses, while MAGEA4-targeted TCR-T cell products, such as afamitresgene autoleucel, have demonstrated promising preliminary efficacy in solid tumor treatment. Recently, the U.S. Food and Drug Administration (FDA) granted accelerated approval for afamitresgene autoleucel (Tecelra, Adaptimmune) as a second-line treatment for metastatic synovial sarcoma, marking a significant milestone for MAGE-A4-directed TCR cancer therapy (Ellis and Weiss 2024). In a clinical trial, 19 out of 44 patients (43%) with metastatic synovial sarcoma exhibited tumor shrinkage, with a median response duration of 6 months (FDA 2024).

To fully harness the potential of MAGEA4-targeted therapies, future research should focus on the following key areas: comprehensive analysis of MAGEA4 expression patterns across various tumor types and their correlation with patient prognosis; experimental validation of proposed immune evasion mechanisms in MAGEA4-positive tumors; and development of strategies to enhance MAGEA4-specific immune responses while counteracting immune evasion tactics. For instance, as MHC-I downregulation is a significant evasion mechanism, exploring combinations of MAGEA4-targeted therapies with drugs that upregulate MHC-I expression may prove beneficial. Similarly, if T cell exhaustion is confirmed as a critical factor, incorporating checkpoint inhibitors into treatment regimens could be advantageous.

In conclusion, MAGEA4, as a highly promising tumor-associated antigen, demonstrates enormous potential in tumor diagnosis, prognosis evaluation, and targeted therapy. Although MAGEA4 shows great promise as a target for tumor immunotherapy, fully realizing its clinical potential requires a deep understanding of the complex immune evasion strategies employed by tumors. As our knowledge of MAGEA4's biological functions deepens and related technologies advance, MAGEA4-based personalized precision treatments hold the promise of bringing new hope to cancer patients.

## Data Availability

No datasets were generated or analysed during the current study.
